# Evaluation of the relationship between dental anxiety and oral health status of mothers and their children

**DOI:** 10.1186/s12903-024-04530-0

**Published:** 2024-06-28

**Authors:** Ekin Besiroglu-Turgut, Sibel Kayaalti-Yuksek, Müge Bulut

**Affiliations:** 1 Department of Periodontology, Faculty of Dentistry, Istanbul Okan University, Istanbul, Turkey; 2 Department of Pediatric Dentistry, Faculty of Dentistry, Istanbul Okan University, Istanbul, Turkey

**Keywords:** Dental anxiety, Oral hygiene, Child, Gingival diseases

## Abstract

**Background:**

Mothers usually have the primary role in raising children and developing health-related behaviors. This study aims to evaluate the relationship between dental anxiety and oral hygiene status of mothers and children’s dental anxiety and gingival health.

**Methods:**

The study included 305 children, aged 4–12 years, who came to the dentist for the first time and their mothers. All the demographic and oral hygiene information were collected through a questionnaire. The dental anxiety of the mothers and children was assessed using the Modified Dental Anxiety Scale (MDAS) and Venham Picture Test (VPT), respectively. The oral examination of the mother and children was performed, and their PI, GI, and DMFT scores were recorded.

**Results:**

While the correlation between MDAS and VPT was positive and strong in children aged 8–12, it was positive but weak in the 4–7 age group. A significant relationship was detected between the mother’s PI, GI, DMFT, and the child’s VPT score. According to the mothers’ dental anxiety, there were no statistically significant differences in PI, GI, and dmft values in children aged between 4 to 7. A moderately positive and statistically significant relationship between maternal dental anxiety and children’s DMFT was identified in children aged 8–12.

**Conclusions:**

Children’s dental anxiety was significantly influenced by maternal dental anxiety, post-treatment complications experienced by the mother, and the oral health status of the mother.

**Trial registration:**

Clinical Trials-ID: NCT05563532; Registration Date: 17.09.2022.

**Supplementary Information:**

The online version contains supplementary material available at 10.1186/s12903-024-04530-0.

## Introduction

Dental anxiety is defined as intense fear and anxiety and is common among children and adults. Direct factors such as age, gender, anxious temperament in childhood, previous traumatic dental experiences, and postoperative complications, as well as indirect factors such as the effect of family on the approach to dental treatment, and sociodemographic factors, have been reported to affect the development of dental anxiety [1, 2]. Dental anxiety is a common concern in the global pediatric population, notably in school and preschool children rather than adolescents [3]. Negative personal experiences or possibly frightening and stereotypical views about dentistry common in today’s popular culture are passed on to children through family members and can cause dental anxiety. Especially parents’ dental anxiety may affect their children’s dental anxiety through modeling or information. Bandura described role modeling, or children’s vicarious identification with their parents, as a form of influence whereby children reproduce the behaviors of their parents through observational and social learning processes [4]. During early childhood, children often emulate their parents, who serve as role models and may integrate their parents’ values and perspectives into their belief systems. Providing information is an essential part of raising a child, especially during their early years. By sharing knowledge and giving instructions, parents can help their children understand and overcome situations that may cause fear. This process of learning and guidance likely plays a significant role in the fears we encounter in our daily lives [2, 5, 6].

Mothers are generally the primary people responsible for child-rearing and developing health-related behaviors in their children. Children tend to internalize their mothers’ values, attitudes, and behaviors [2]. The mother’s oral health-related behaviors, such as oral care habits, dental anxiety, and dental attendance patterns, influence the child’s dental care efforts, frequency of toothbrushing, behavior in a dental setting, dental anxiety, and dental visits [5]. In accordance with the findings of Gottems et al., maternal dental anxiety exerts an influence not only on children’s dental anxiety, but also on the frequency of tooth brushing [5]. When mothers postpone dental visits due to their own concerns, it can disrupt the routine dental visits for their children as well. Delayed dentist visits can postpone the establishment of proper oral hygiene habits and lead to missed opportunities for preventive care and early intervention for dental problems [7, 8]. Maternal dental anxiety has been suggested as an indicator of children’s oral health [9]. For these reasons, as evidenced by studies, children of highly anxious mothers tend to have the highest rates of decay, and missing or filled teeth [7].

Childhood is a critical period during which knowledge, behaviors, and habits that can influence oral health are acquired. The overall health of adults is largely determined by the dental care habits developed during childhood. Behaviors and experiences related to oral health play a role in establishing and accepting oral hygiene practices during childhood [5]. Because dental anxiety leads to the avoidance of dental procedures, including tooth brushing, studies have found that children with high levels of dental anxiety tend to have higher values of dental plaque and gingival index compared to children with low anxiety [10, 11].

Studies investigating the influence of mothers on children’s oral health and dental anxiety remain limited and lacking in clarity, therefore necessitating further research. The topic of anxiety about dental treatment needs to be researched in different populations, to minimize the impact of maternal factors on oral treatments and oral health.

The principal objective of this investigation is to examine the association between maternal dental anxiety and children’s dental anxiety levels and gingival health. The secondary objective of this research is to assess the correlation between the oral hygiene status of mothers, the dental anxiety levels of children, and the gingival health of children. The study hypothesizes that children of mothers who have high dental anxiety have increased dental anxiety and poor gingival health. Furthermore, the dental anxiety and gingival health of children of mothers with inadequate oral care habits are negatively affected.

## Methods

This cross-sectional study was conducted on children and their mothers who applied to Istanbul Okan University, Faculty of Dentistry, between 2019–2023. The study protocol was approved by the Ethics Committee of Istanbul Okan University (2019/104.25). Children between the ages of 4–12, without any known systemic diseases, who are visiting a dentist for the first time and do not have acute pain complaints, and mothers with at least 20 teeth who do not have any systemic or psychiatric disorders, are not pregnant or breastfeeding, do not smoke, and have not received periodontal treatment in the past six months were included. The children were also evaluated in the age groups of 4–7 and 8–12.

Before commencing the study, the participants received an explanation of the project from MB. A written informed consent form was obtained from the mothers who agreed to participate in this study with their children. The sample size was calculated with G*Power 3: two-tail; α = 0.05; power (1-β) = 0.8 [12]. A total of 350 mother–child pairs were initially recruited in the study, but after the exclusion of 45 mothers who did not fully complete the questionnaire, the evaluations were made of 305 mother–child pairs.

The Venham Picture Test (VPT) was applied to the children, and the Modified Dental Anxiety Scale (MDAS) was applied to the mothers to determine dental anxiety levels. A questionnaire was administered to the mothers to evaluate sociodemographic data, oral health awareness, oral care habits, and previous dental experiences. All the surveys were applied, and demographic data were recorded by the same researcher (MB). The MDAS consists of 5 items providing a total score ranging from 5 to 25 points. MDAS score ≥ 19 is evaluated as a high level of dental anxiety. Ilguy et al. conducted a Turkish validity and reliability study of the MDAS in 2005, determining the Cronbach alpha value to be 0.81 [13].

The VPT consists of eight illustrations of human figures expressing various emotional reactions. The child selects pictures to reflect their current emotions. Each picture of an anxious child is assigned a score of 1, and each picture of a non-anxious child is assigned a score of 0. The total score ranges from 0 to 8, with a high score indicating a high level of anxiety, and a score of ≥ 4 is evaluated as anxious [14]. The children were separated into two groups based on whether they were classified as anxious. The anxious group comprised 145 children, and the non-anxious group had 160 children.

First, children were allowed to complete the VPT. After mothers filled out anxiety forms and questionnaires (related to educational level, demographic characteristics, oral hygiene habits, and previously experienced complications) regarding dental treatments, a single researcher (EBT), who was blinded to the data, performed oral examinations first on the children and then on the mothers. Subsequently, periodontal examinations of the four surfaces (mesial-distal-lingual-buccal) of all the teeth were performed with a William periodontal probe, while dental examinations were performed with a curved probe. Dental caries among children and mothers was diagnosed using the World Health Organization (WHO) criteria. The decayed, missing, and filled teeth (dmft/DMFT) were calculated for each individual [15]. The Silness Löe plaque index [16] (PI) was assessed by running a periodontal probe parallel along the gingival margin and was scored from 0 to 3 (0: free of plaque, 1: visible plaque with the probe, 2: visible plaque with the naked eye, 3: accumulation of heavy plaque). The Löe Silness gingival index [17] (GI) was used to evaluate gingival inflammation and was scored from 0 to 3 (0: healthy gingiva, 1: mild inflammation and edema, no bleeding on probing, 2: moderate inflammation and edema, bleeding on probing, 3: severe inflammation and tendency to spontaneous bleeding). The measurements in children were conducted using distraction techniques (such as watching cartoons) and the tell-show-do method under the supervision of a pediatric dentist [18]. The total plaque and gingival scores were calculated by adding the scores of all the surfaces of each tooth and dividing that by the number of surfaces. The intra-examiner reliability and repeatability were assessed with the intraclass correlation coefficient. The measurements were made twice at one-week intervals in a separate sample of 10 patients, and intra-examiner reliability and repeatability were assessed using the intraclass correlation coefficient.

### Statistical analysis

Descriptive statistics for the data were calculated. Before proceeding with the hypothesis tests, continuous variables were examined with the Shapiro–Wilk test for compliance with normal distribution, one of the parametric test assumptions, and with the Levene test for homogeneity of variances. Since the data did not meet parametric test assumptions, the Mann–Whitney U test was used for pairwise group comparisons, and the Kruskal–Wallis test was used for comparisons of more than two groups. When comparing more than two groups, the Dunn-Bonferroni test was used for post-hoc testing after detecting a significant difference. The *p* < 0.05 criterion was used in statistical evaluations. The Stata 18 software was used for all analyses.

## Results

Evaluations were made on 305 children, comprising of 164 boys and 141 girls, and their respective mothers. Among these children, 180 fell within the age range of 4 to 7 years, while the remaining 125 were between 8 and 12 years old. The mothers’ ages ranged from 25 to 51 (mean ± SD 36.61 ± 6.19). Of the mothers participating in the study, 123 (40.3%) were primary school graduates, 62 (20.3%) were high school graduates, 93 (30.5%) were university graduates, and 27 (8.9%) had completed postgraduate degrees.

While the correlation between MDAS and VPT was positive and strong in children aged 8–12 (*p* < 0.001, r_s_ = 0.476), it was positive but weak in the 4–7 age group (*p* < 0.001, *r* = 0.282). Specifically, among children aged 4–7, those whose mothers exhibit higher MDAS scores tend to have higher VPT scores (*p* < 0.001) (Table 1). In contrast, when evaluating VPT scores in children aged 8–12 based on their mothers’ MDAS scores, the differences were not statistically significant (*p* = 0.213). The comparison of the child’s dental anxiety, based on the complications experienced by the mothers during their previous dental treatments, is provided in Table 2. Based on maternal education level, it was observed that the VPT scores were highest among children whose mothers had postgraduate education, whereas children of mothers with primary education displayed the lowest scores(*p* < 0.05).

**Table 1:** Relationship of VPT values with MDAS, Mothers’ PI, Mothers’ GI and Mothers’ DMFT values according to age groups

		**Children VPT**	
		**Children age group**	
		***4–7 age(n* = *180)***	***8–12 age(n* = *125)***
**Mother PI**	**r**_**s**_	0.513	0.373
	***p***	< 0.001	< 0.001
**Mother GI**	**r**_**s**_	0.364	0.286
	***p***	< 0.001	0.001
**Mother DMFT**	**r**_**s**_	0.564	0.206
	***p***	< 0.001	0.021
**MDAS**	**r**_**s**_	0.282	0.476
	***p***	< 0.001	< 0.001

r_s_: Spearman Rho Correlation, significance level *p* < 0.05

**Table 2:** Comparison of children’s VPT value according to the mother’s previous dental complications

	**Child VPT**		
	**Mean ± S. Deviation**	**Median (Min.—Max.)**	***p***
**Having problems at Previous dental treatments**			
**No**	2.33 ± 0.58	0 (0—7)	0.032 y
**Yes**	1.15 ± 0.27	0 (0—8)	
**Complication at Previos Dental Treatments**			
**Pain**	1.74 ± 0.33	0 (0—8) c	< 0.001 x
**Swelling**	3.28 ± 0.3	0 (0—8) b	
**Bleeding**	8 ± 0	8 (8—8) a	
**Pain and Swelling**	5.58 ± 0.47	7 (0—7) b	

x: Kruskal Wallis H test, y: Mann–Whitney U test, a-c: there is no difference between groups with the same letter, and data were given as median (min–max), significance level *p* < 0.05

A strong, positive, and statistically significant relationship was found between the mother’s PI and the child’s VPT score. A moderately strong, positive, statistically significant relationship was found between the mother’s GI and the child’s VPT score. While the relationship between the mother’s PI and DMFT values and child’s VPT score was strong in children aged 4 to 7 (*p* < 0.001, r_s_ = 0.373), it was weak in children aged 8 to 12 (*p* = 0.021, r_s_ = 0.206) (Table 1). There was no statistically significant relationship between the mother’s oral hygiene habits and the child’s dental anxiety (*p* = 0.477).

There was no statistically significant relationship between the mother’s brushing habits and the children’s PI and GI values (*p* = 0.437, *p* = 0.685, respectively). According to the mothers’ dental anxiety, there were no statistically significant differences in PI, GI, and dmft values for the children aged 4 to 7 (*p* < 0.05, r_s_ = 0.164, r_s_ = -0.091, r_s_ = -0.019, respectively) Only a moderately positive and statistically significant relationship between maternal dental anxiety and children’s DMFT was identified in children aged 8 to 12 (r_s_ = 0.45, *p* < 0.001) (Table 3).

**Table 3:** Relationship (correlation) between mothers’ MDAS and childrens’ PI, GI and DMFT values

		**MDAS**	
		**Children age group**	
		**4–7 age (*n* = 180)**	**8–12 age (*n* = 125)**
**Children PI**	r_s_	0.164	0.216
	*P*	0.028	0.015
**Children GI**	r_s_	-0.091	0.161
	*P*	0.223	0.074
**Children dmft**	r_s_	-0.019	-0.227
	*P*	0.802	0.011
**Children DMFT**	r_s_	0.02	0.45
	*P*	0.793	< 0.001

r_s_: Spearman Rho Correlation, significance level *p* < 0.05

The relationship between children’s VPT values and children’s PI, GI, and dmft values is shown in Table 4. Moderate and statistically negative correlations were found between children’s age and VPT score (r_s_ = -0.527; *p* < 0.001). Notably, there were no statistically significant differences in VPT scores based on the children’s gender (*p* = 0.214).

**Table 4:** Relationship between childrens’ VPT values and childrens’ PI, GI, DMFT values

	Children VPT	
	r_s_	*p*
Children PI	0,553	< 0,001
Children GI	0,500	< 0,001
Children dmft	0,291	0,041
Children DMFT	-0,175	0,225

r_s_: Spearman Rho Correlation, significance level *p* < 0.05

It was determined that PI, GI, and DMFT values were higher in children aged 4–7 compared to those aged 8–12 (*p* < 0.001). Only the DMFT score was found to be greater in the 8–12 age group than in the 4–7 age group (*p* < 0.001).

## Discussion

Dental anxiety, which can influence oral hygiene habits, is a personality trait with several origins [19]. According to research, traumatic experiences and social learning are the primary sources of dental anxiety [5, 6]. Maternal figures, who play a crucial role in social learning, are typically the primary caregivers responsible for nurturing and shaping health-related behaviors in their children. Given that children spend a considerable amount of time with their mothers, they are inclined to mimic their attitudes and behaviors [20]. This study encompasses two objectives: (1) to investigate the relationship between maternal dental anxiety and the dental anxiety levels and gingival health of children, and (2) to evaluate the connection between the oral hygiene status of mothers and the dental anxiety levels and gingival health of their children. Furthermore, this study underscores the importance of targeting social behaviors to enhance oral health maintenance and awareness.

Based on the outcomes of the present investigation, it can be concluded that as the dental anxiety level of the mothers increased, the dental anxiety level of their children increased. There are studies in the literature reporting that mothers’ anxiety influences children’s anxiety levels [7, 12, 21]. In a sample of Finnish children, Rantavuori et al. [22] discovered intergenerational transmission of dental anxiety. Their findings indicated that individuals with a parent who exhibited dental anxiety were more inclined to report experiencing dental anxiety themselves, in comparison to individuals whose parents did not display dental anxiety. Busato et al. [12] showed that maternal anxiety had an effect of 81.3% on child anxiety. Majstorovic et al. [23], stated that having a parent with an MDAS score > 15 increases the likelihood of dental anxiety in children by approximately 150% when compared to parents with lower scores. However, these results differ from some published studies showing that children’s anxiety is not influenced by the mother’s dental anxiety [24, 25] These conflicting results could be associated with the child’s age group and gender, visiting the dentist for the first time, experiencing a traumatic treatment, and sociodemographic factors. Age is thought to play a significant role in dental anxiety. Generally, the influence of mothers on dental anxiety is more common in younger children [26], particularly those under the age of 8 years [27]. In our study, this relationship was stronger in children aged 8–12, it was weaker in children aged 4–7. Similar to our study, Assunçao et al. in Brazil found a positive association between children aged 8–11 years and the mother’s dental anxiety [28]. Young children fear the unknown and abandonment. It is thought that young children do not perceive the fearful situation clearly due to their lack of cognitive ability and are affected by their parents’ fear and anxiety [29]. Conversely, older children, with higher levels of attention and cognition, can internalize their family’s experiences by attributing them to causal factors in their own lives [12]. Further research could delve deeper into understanding the cognitive and emotional processes underlying these relationships.

According to Koenigsberg and Johnston, the maternal dental anxiety effect is only present until the child’s first visit, after which “previous experience” has a greater weighting [30]. Negative attitudes and experiences transmitted by mothers have been considered to be etiological factors of dental anxiety in children [31, 32]. The current study, which found that children whose mothers had bleeding complications had the highest levels of anxiety, not only confirms this information but also demonstrates that mothers of children who have had complications after previous dental treatments tend to have higher levels of anxiety. One of the strengths of this study is that it compared the dental anxiety levels of the children that had not been evaluated before with the complications from the mothers’ previous dental treatments. Recently, some evidence has emerged regarding the effectiveness of observing models in the management of anxiety in pediatric dental patients. Some authors have focused on the principles of social learning and discovered that exposing children to positive images reduces dental anxiety [33]. Based on this, it can be postulated that dental complications experienced by mothers, particularly those resulting in visible alterations, might likewise influence the dental anxiety of their children. However, longitudinal studies are necessary to ascertain the impact of complications experienced by mothers after dental treatments on the dental anxiety of their children.

Our study showed that mothers’ oral health indicators significantly influenced children’s dental anxiety, particularly among children aged 4 to 7 years old. Anxiety can emerge in young children in response to events and changes in their immediate environment. Children have a tighter bond with their mothers at a young age, therefore it is likely that they will closely watch and be impacted by changes in their mothers [34]. Based on this, it is hypothesized that young children’s dental anxiety may be influenced by observing gingival bleeding and sensitivity in mothers with a high plaque index or by witnessing closely the mother’s dental pain in mothers with a high number of cavities. However, to establish a causal relationship, future studies could be recommended to explore additional variables, such as the mother’s character traits, patterns in the mother–child relationship, and environmental factors.

Parents influence their children’s values, perspectives, and behavior related to dental care through various means in their daily lives, both through their conduct and through direct instruction regarding the significance of dental care [27, 35, 36]. Mothers who adopted unhealthy behavioral patterns, such as irregular dental check-ups and low brushing frequency, were usually less likely to set an example and take good care of their children’s oral health [37]. Okada et al. [38] found that parents’ attitudes towards oral care had a significant positive effect on their children’s dental health, including caries and gingival health. A Japanese study argued that gingival condition rather than DMFT is a better parameter for present oral care. This study found a link between a mother’s gingival condition and her child’s caries prevalence and severity at age three. In another study conducted by Okada et al. [39], it was discovered that the gingival health of mothers had a direct impact on their children’s gingival health and susceptibility to cavities. Furthermore, researchers discovered that the worse a mother’s gingival health, the less likely her child would be caries-free. A bivariate analysis was conducted among 457 mother–child pairs in Tehran, which showed a positive relationship between mothers’ oral health knowledge, attitudes, and their children’s dental health [40]. Despite the prevailing findings, our study did not discern a direct association between maternal oral care and children’s oral health outcomes. Nonetheless, it is conceivable that an indirect influence may exist, potentially mediated through the modulation of dental anxiety. Further research is needed to explore the underlying mechanisms and to develop strategies for promoting better oral care practices among both parents and children, ultimately aiming to improve the oral health and overall well-being of future generations.

Several studies have shown that mothers’ dental anxiety is a risk factor for dental caries in their children [41–43]. Khawja et al. reported that an association was observed between high anxiety levels in mothers and an increased incidence of decayed and missing teeth in children aged 3–14 years [8]. In a study conducted on 556 mother–child pairs, it was found that the dental anxiety of mothers is not only a factor accompanying the development of dental anxiety in children, but also affects their oral health [35]. According to Gottems et al., mothers’ oral health-related actions do not directly impact the oral health of their children but have an indirect effect through their effect on the child’s teeth brushing and dentist attendance [5]. The possible explanation for these disparities between studies is that the mother–child relationship can be affected by the mother’s personality, family dynamics, and the social environment of the family. Despite the diverse findings within the existing literature, recent research has elucidated that the influence of children’s dental anxiety on their oral health surpasses that of maternal dental anxiety [35]. In line with this observation, our present study did not reveal any significant correlation between maternal dental anxiety and the oral health status of their children. Nevertheless, a noteworthy discovery in our investigation pertains to the higher prevalence of dental plaque, gingival inflammation, and dental caries among children exhibiting elevated levels of dental anxiety. Folayan et al. [44] found that children with low dental anxiety had poorer oral hygiene and more calculus and plaque when they evaluated 450 children aged 6 to 12 years. In a study conducted on children aged 2–5 years, it was determined that high dental anxiety levels were directly proportional to periodontal index scores [43]. Colares and Richman [45] reported that all children with negative behavior had dental caries, with the majority having a dmft score > 8. A recent study found that PI and GI values were significantly higher in anxious children [11]. One should bear in mind that childhood is a crucial period for gaining knowledge and habits. The possibility that dental anxiety may affect brushing habits should be carefully evaluated by clinicians.

According to the findings of this study, PI, GI scores, and dental anxiety in children decrease with age. Folayan et al. reported that the level of dental anxiety began to decrease around the age of 6–7 years, and the ability to cope with dental treatments increased as the age progressed [2]. Another study, however, found that psychological development had a greater effect than chronological age in reducing dental anxiety [46]. Pinkham claimed that after the age of four years, many children have reached a level of cognitive maturity that allows them to manage their anxiety [47]. Therefore, the importance of age in dental anxiety is not surprising, as age reflects different aspects of psychological development. In the dual-cohort cross-sequential study conducted by Storman et al., dental caries rates were found to be similar between different periods and cohorts at the end of the follow-up period in the birth group and kindergarten group, while the highest caries rate was observed in the 8-year-old group. Furthermore, the study revealed a progressive increase in daily brushing rates from the ages of 2 to 6 [48]. A possible explanation for this might be that the development of oral hygiene practices with age may contribute to the observed decrease in PI and GI scores with increasing age.

There were certain limitations to this study. In the present study, anxiety levels were determined using a projective test (VPT) for children and a psychometric test (MDAS) for mothers. Physiological measurements, such as pulse rate, oxygen saturation, and blood pressure, could also be employed to evaluate anxiety. Although we chose not to use physiological measurements, which could potentially induce additional stress in children, this area could be fruitful for further research. Indices to determine the character traits of mothers were not included in our questionnaire form. However, for future studies, it is recommended to assess the influence of maternal character traits on children, as this would provide a more comprehensive understanding of the issue. Given the multifactorial etiology of dental anxiety, it can be advised to conduct more comprehensive studies encompassing variables such as maternal and child characteristics, diverse sociocultural conditions, and the involvement of other family members. This cross-sectional study could not examine whether dental anxiety in this population predicts future improved oral health habits and how it may evolve. In addition, the study did not include non-clinical populations. Hence, further longitudinal research needs to be conducted to investigate the clinical and non-clinical populations.

## Conclusion

Based on this study’s results, the following conclusions can be drawn:

1. The current study, within its limitations, observed a significant impact of mothers’ dental anxiety on children’s anxiety levels. The association between mothers’ and the children’s dental anxiety varied according to age group, with a strong and positive correlation between MDAS and VPT observed in children aged 8–12, whereas it was positive but weak in the 4–7 age group.
2. Although dental complications experienced by mothers and their oral health status significantly impacted children’s anxiety levels, no significant relationship was found between the mother’s oral hygiene habits and the child’s dental anxiety.
3. While no relationship was observed between mothers’ dental anxiety and brushing habits and the oral health of children aged 4–7, a significant association was found between mothers’ anxiety and DMFT scores, specifically in children aged 8–12.
4. The difference between the children’s age and anxiety scores was significant, however, there was no difference observed between the children’s gender and anxiety scores.

### Supplementary Information

Additional file 1. The questionnaire for this survey.

## Data Availability

The datasets generated during and/or analyzed during the current study are available from the corresponding author upon reasonable request.

## Abbreviations

MDAS: Modified Dental Anxiety Scale
VPT: The Venham Picture Test
PI: The Silness Löe plaque index
GI: The Löe Silness gingival index
DMFT: The decayed, missing, and filled teeth
dmft: The decayed, missing, and filled teeth for temporary tooth
WHO: World Health Organization
