# Supplementary material for: Evaluation of the relationship between dental anxiety and oral health status of mothers and their children
Source: BMC Oral Health. 2024 Jun 28;24:749. doi: 10.1186/s12903-024-04530-0 (PMC11212381; doi:10.1186/s12903-024-04530-0)
Supplement: Supplementary file 1 — Additional file 1. The questionnaire for this survey. [file 12903_2024_4530_MOESM1_ESM.docx]

**Date:**

**QUESTİONNAİRE FORM**

The purpose of applying this form is to determine the mother's oral health habits and dental anxiety and evaluate their effects on her children.

**Mother’s Age: Child’s Age:**

**Education level:** Primary education High school University Master'/Ph.D.

**Family's Monthly Income:** <2250 2250-5000 >5000

**1.** **What is your daily toothbrushing frequency?**

a) Does not brush and/or is uneven

b) 1 per day

c) 2 and/or more per day

**2. Do you use any additional maintenance tools? Tick the ones you use.**

a) Floss

b) Toothpick

c) Interdental brush

d) Mouthwash

**3. How often do you go to the dentist?**

a) When you have a complaint

b) Occasionally

c) Regularly

d) I'm coming for the first time

**5.** **Have you had any problems with your previous dental treatments?**

a) Yes

b) No

**6.** **What complications did you experience after your previous dental treatments?**

a) Pain

b) Swelling

c) Bleeding

**Modified Dental Anxiety Scale**

The following questions have been prepared to determine your fear and anxiety about the dentist and/or the treatment you will receive. Please select the option that best suits you.

**1) If you went to your dentist for treatment tomorrow, how would you feel?**

a) Not anxious

b) Slightly anxious

c) Fairly anxious

d) Very anxious

e) Extremely anxious

**2) If you were sitting in the waiting room (waiting for treatment), how would you feel?**

a) Not anxious

b) Slightly anxious

c) Fairly anxious

d) Very anxious

e) Extremely anxious

**3) If you were about to have a tooth drilled, how would you feel?**

a) Not anxious

b) Slightly anxious

c) Fairly anxious

d) Very anxious

e) Extremely anxious

**4) If you were about to have your teeth scaled and polished, how would you feel?**

a) Not anxious

b) Slightly anxious

c) Fairly anxious

d) Very anxious

e) Extremely anxious

**5) If you were about to have a local anesthetic injection in your gum, above an upper back tooth, how would you feel?**

a) Not anxious

b) Slightly anxious

c) Fairly anxious

d) Very anxious

e) Extremely anxious
